# Risk factors for mortality among adults registered on the routine drug resistant tuberculosis reporting database in the Eastern Cape Province, South Africa, 2011 to 2013

**DOI:** 10.1371/journal.pone.0202469

**Published:** 2018-08-22

**Authors:** Ruvimbo Chingonzoh, Mohamed R. Manesen, Mncedisi J. Madlavu, Nokuthula Sopiseka, Miyakazi Nokwe, Martin Emwerem, Alfred Musekiwa, Lazarus R. Kuonza

**Affiliations:** 1 School of Health Systems and Public Health, Faculty of Health Sciences, University of Pretoria, Pretoria, South Africa; 2 South African Field Epidemiology Training Programme, National Institute for Communicable Diseases, National Health Laboratory Service, Johannesburg, South Africa; 3 Provincial Epidemiology Team, National Institute for Communicable Diseases, National Health Laboratory Service, Johannesburg, South Africa; 4 HIV and AIDS, STIs and TB (HAST) Programme, Eastern Cape Department of Health, King Williams Town, South Africa; 5 Amity Health Consortium, Johannesburg, South Africa; University of Cape Town, SOUTH AFRICA

## Abstract

**Introduction:**

South Africa is among countries with the highest burden of drug resistant tuberculosis (DR-TB). The Eastern Cape Province reported the highest MDR-TB mortality rates in South Africa for the 2010 treatment cohorts. This study aimed to determine risk factors for mortality among adult patients registered for DR-TB treatment in the province.

**Methods:**

We conducted a retrospective cohort study of adult patients treated for laboratory confirmed DR-TB between January 2011 and December 2013. Demographic and clinical characteristics of the patients were obtained from a web-based electronic database of patients treated for DR-TB. We applied modified Poisson regression with robust standard errors to identify risk factors for DR-TB mortality. We also stratified the analyses into multi-drug resistant TB (MDR-TB) and extensively drug resistant (XDR-TB).

**Results:**

Among 3,729 patients that met the inclusion criteria, 39% (n = 1,445) died. Of the patients that died, 53% (n = 766) were male, 68% (n = 982) had MDR-TB, 72% (n = 1,038) were HIV co-infected, and median age was 37 years (Interquartile Range [IQR] 30–46). Patients were at higher risk of mortality during DR-TB treatment if they were HIV co-infected not on antiretroviral treatment (ART) (adjusted incidence risk ratio [aIRR] 3.3, 95% confidence interval [CI] 2.9–3.8), were 60 years or older (aIRR 1.7, 95%CI 1.5–2.0), had a diagnosis of XDR-TB (aIRR 1.6, 95%CI 1.5–1.7), or had been hospitalised at treatment start (aIRR 1.7, 95%CI 1.5–1.8). Among MDR-TB patients, risk of mortality was higher if patients were HIV co-infected not on ART (aIRR 3.9, 95%CI 3.3–4.6), were 60 years or older (aIRR 1.9, 95%CI 1.6–2.3), or had been hospitalised at start of MDR-TB treatment (aIRR 1.7, 95%CI 1.5–1.9). Among XDR-TB patients, risk of mortality was higher in patients who were HIV co-infected not on ART (aIRR 1.8, 95%CI 1.5–2.2), or had been hospitalised at the start of XDR-TB treatment (aIRR 1.5, 95%CI 1.3–1.8).

**Conclusion:**

HIV co-infected not on ART, older age, XDR-TB and hospital admission for DR-TB treatment were independent risk factors for DR-TB mortality. Integration of TB and HIV services, with focus on voluntary HIV testing and counselling of DR-TB patients with unknown HIV status, and provision of ART for all co-infected patients may reduce DR-TB mortality in the Eastern Cape.

## Introduction

Tuberculosis (TB), a preventable and curable disease, remains one of the leading causes of death globally [1]. The World Health Organization (WHO), through the End TB Strategy [2], envisions the eradication of death, disease and suffering due to TB by 2035 [1]. Drug resistant TB (DR-TB) is an impediment to realising this vision [3] because of lengthy [3], toxic [4], more costly treatment [5], and poorer treatment outcomes [6] when compared to drug susceptible TB.

DR-TB can be classified as multi-drug resistant TB (MDR-TB) or extensively drug resistant TB (XDR-TB). MDR-TB is defined as TB where there is in-vitro resistance to both isoniazid and rifampicin and XDR-TB is MDR-TB with additional in-vitro resistance to any fluoroquinolone and any of the three second line injectable anti-TB drugs [7, 8].

Globally, over 153,119 MDR-TB cases and 8,014 XDR-TB cases were reported to the WHO in 2016 [1]. Among 30 high TB burden countries, South Africa ranked third highest (n = 19,073) after India (n = 37,258) and Russia (27,363) in MDR-TB notifications and third highest (n = 967) in XDR-TB notifications following India (n = 2,464) and Ukraine (1,195) [1].

In South Africa, patients who screen positive for TB submit sputum for GeneXpert MTB/RIF (GXP) testing. GXP detects the presence of *Mycobacterium tuberculosis*, and determines its susceptibility or resistance to rifampicin [1, 7]. On receipt of the results, the diagnosing facility, usually a community based primary health care facility, traces the patient for follow-up testing and treatment initiation. Patients diagnosed with rifampicin susceptible TB are treated at the facility of diagnosis [7, 9], or referred to a primary health care facility that is convenient to them. Patients exhibiting TB that is resistant to rifampicin are referred to a DR-TB facility for further evaluation and management. Such patients will submit a second sputum sample for definitive diagnosis of DR-TB through drug susceptibility testing (DST) or Line Probe Assay (LPA) [7]. Patients who test GXP positive for TB with rifampicin resistance are classified as having laboratory confirmed MDR-TB if the organism exhibits resistance to first line drugs including isoniazid on conventional DST test and or LPA [7, 10]. Organisms shown to be resistant to rifampicin and or isoniazid will be tested for resistance to second line anti-TB drugs [7].

All patients diagnosed with rifampicin resistant tuberculosis (with or without additional drug resistance) are entered into a paper based treatment register which is then transcribed onto an electronic register. Patients that exhibit rifampicin resistance on GXP are registered and started on MDR-TB treatment. On receipt of laboratory confirmation results, resistance profile is reviewed, DR-TB type determined and the DR-TB patient register is updated [7]. Before introduction of shortened DR-TB regimens in 2017, treatment for DR-TB extended from 24 to 36 months [7, 10–14].

Among patients, in South Africa, started on DR-TB treatment in 2013 (2013 DR-TB treatment cohort), 23.6% (n = 2,707) died. Across all nine provinces in the country, the Eastern Cape Province reported the highest proportion of deaths for the 2013 DR-TB treatment cohort (37.1%, n = 816) [15]. For the 2010 treatment cohort South Africa reported MDR-TB mortality rates of 17%, with the Eastern Cape Province reporting the highest MDR-TB mortality rate of 27.7% [16]. South Africa reported high XDR-TB mortality rates which ranged from 47% [17] for the 2012 treatment cohort and 42% [1] for the 2014 treatment cohort.

The high XDR-TB mortality in South Africa is associated with high rates of Human Immunodeficiency Virus (HIV) co-infection [1, 18]. According to a national study conducted among patients who started treatment between 2009 and 2011, 53% (n = 9,419) of DR-TB patients included in the study were co-infected with HIV [10]. One South African study determined that 59% of XDR-TB patients who started treatment between 2013 and 2014, were HIV co-infected [19]. Kvasnovsky and colleagues determined that, in the Eastern Cape, HIV co-infection rate among XDR-TB patients, who started treatment between 2006 and 2008, was 56% [20], while another study conducted in North West Province, South Africa determined MDR-TB HIV co-infection rate, among patients started on treatment between 2000 and 2008, to be 59%. The North West Province study also determined that provision of anti-retroviral therapy (ART) in HIV co-infected persons was protective for TB associated mortality [21]. In South Africa, HIV counselling and testing is offered to DR-TB patients and all HIV co-infected DR-TB patients are eligible for ART initiation irrespective of CD4 cell count [7, 22].

Understanding risk factors for DR-TB mortality is vital to improving DR-TB treatment outcomes. Previous studies have shown that increasing age [23, 24], comorbidities [20, 25] such as diabetes and HIV, as well as anti-TB treatment history [25] are risk factors for unfavourable DR-TB treatment outcomes.

This study aimed to utilise routine DR-TB patient data to determine factors contributing to DR-TB mortality in the province. Determining risk factors associated with mortality will guide the development of interventions to improve DR-TB treatment outcomes in the province. The objectives of this study were to describe the epidemiology of DR-TB mortality and to determine associated risk factors among adults registered for treatment in the Eastern Cape Province, South Africa.

## Methods

### Study design and setting

We conducted a retrospective cohort study using secondary data extracted from the Electronic Drug-Resistant Tuberculosis Register (EDRWeb). The EDRWeb is a web-based electronic database of patients on DR-TB treatment that has been maintained by the South African National TB Programme since 2009. [10].

According to the 2017 mid-population estimates released in July 2017, the Eastern Cape Province has the third largest population of 6,498,700 accounting for 12% of the country’s population [26]. Approximately 60% of the population live in rural areas [27]. The province has the highest unemployment [28] and second highest poverty levels in the country [29]. In 2014 HIV/AIDS and TB were the leading causes of death in the province [30]. The province has two specialized DR-TB hospitals that manage both MDR- and XDR-TB patients, and eight decentralised MDR-TB facilities equipped to manage MDR-TB patients. Decentralisation of MDR-TB services in the province began in 2011 [31]. According to the South African DR-TB guidelines [7], clinically unstable MDR-TB patients, patients with extensive disease, as well as XDR-TB patients require admission for DR-TB treatment while ambulant patients, who are in fair to good condition and are smear negative should be initiated on ambulatory care [16, 32].

### Study population

We included all adult patients, aged 18 years and older, who had laboratory confirmed DR-TB, were registered on EDRWeb and started treatment between January 2011 and December 2013. Patients were followed up for at least 24 months from treatment start, with follow-up data censored at 31 December 2015. According to the national DR-TB guidelines, patients are assigned treatment outcomes at the end of DR-TB treatment (usually 24 months) [7]. Patients who had mono or poly resistant TB, or had not been assigned a treatment outcome at the end of the follow-up period, were excluded from the analysis. Mono-resistance is defined as resistance to a single first-line anti-TB drug, and poly-drug resistance is resistance to two or more anti-TB drugs excluding rifampicin or isoniazid [7].

### Variables

Death, as defined by the National TB Programme, was the main outcome of this study. The South African National TB Programme, in accordance with WHO classification, defines the treatment outcome “died” as any patient who dies during the course of DR-TB treatment [1]. Following international guidelines [33] and national policies [7], DR-TB was stratified into MDR- and XDR-TB according to the antimicrobial resistance pattern exhibited on drug susceptibility testing. Resistance pattern was reviewed in as far as to assign DR-TB type based on laboratory confirmation test results [7]. Independent variables considered in the analysis included demographic (age, sex, district, anti-TB drug history, treatment start site) and clinical (HIV status, ART initiation status) variables. Data on severity of disease was limited with some variables (e.g. cavitation on chest radiography, comorbidities other than HIV) not available on the electronic patient register, and others (e.g. CD4 count, smear grade) available, but poorly populated. Information on site of treatment start (admission in a DR-TB facility versus ambulatory or outpatient treatment) is routinely captured on EDRWeb.

### Data management

De-duplication of data was done using probability record linkage techniques with variables including name, age, and address of patients. Duplicates were managed by retaining the most recent TB episode, updating anti-TB drug history if the patient had been on treatment for more than one month, for the preceding TB episode, as prescribed in the national DR-TB guidelines [7] and excluding previous partial registrations. Data cleaning and de-identification was done before analyses were conducted using STATA version 13 (StataCorp. College Station, TX, USA). Variables included in the analysis had less than 5% missing data, and the missing data was managed through STATA based, available case analysis.

### Data analysis

Age, in years, was summarised using median and interquartile range (IQR) since it was not normally distributed. Categorical variables were described by proportions and frequency distributions and compared using the Pearson’s Chi-square test. Risk factors for mortality were estimated using modified Poisson regression with robust standard errors to produce incidence risk ratios (IRR) which were reported with the corresponding 95% confidence intervals. Variance inflation factor (VIF) was used to check for multi-collinearity between variables. Variables with VIF values greater than 10 were investigated further and managed by omitting one of the highly correlated variables from the model, or merging the variables into one. Hosmer-Lemeshow Chi-square test was used to determine goodness of fit of the regression model.

### Ethical considerations

After de-duplication, the dataset was anonymised and stored on password protected computers. This study was approved by the Faculty of Health Sciences Research Ethics Committee of the University of Pretoria (reference number 454/2015), and the Eastern Cape Provincial Department of Health Research Committee (reference number EC_2015RP54_763).

## Results

**Fig 1:**
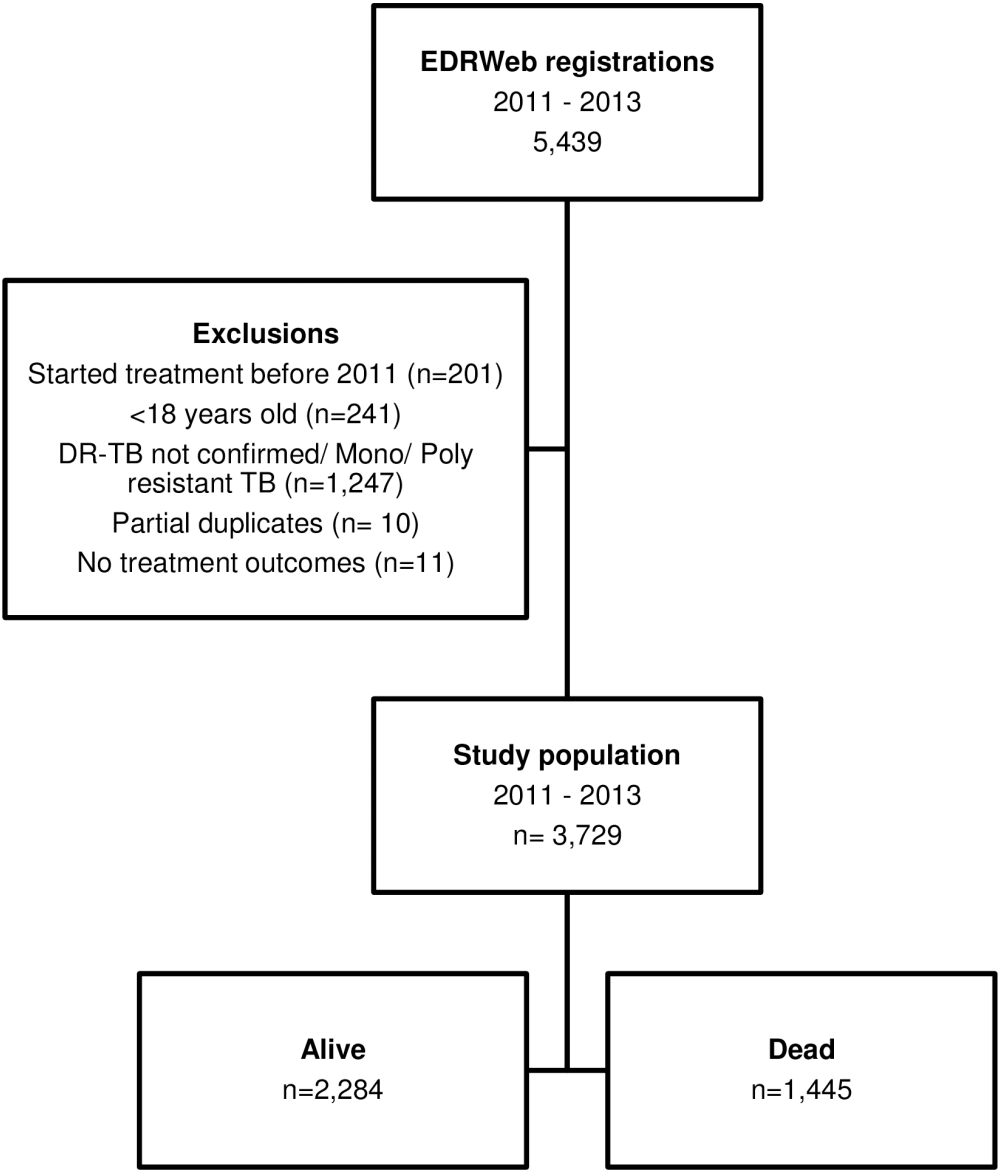
Inclusion and exclusion of DR-TB study population, Eastern Cape Province, 2011–2013. DRT-TB, drug resistant tuberculosis.

A total of 5,439 patients were registered for DR-TB treatment between 2011 and 2013. We analysed data from 3,729 patients with laboratory confirmed MDR- or XDR-TB, after excluding patients who were less than 18 years of age (n = 241) and patients who had mono or poly resistant TB or no DR-TB confirmation (n = 1,247). We excluded 201 patients who were registered during the study period but had been started on treatment prior to 1 January 2011. We also excluded 10 partial duplicate registrations and 11 patient registrations with no treatment outcomes (Fig 1).

### Characteristics of study population

Out of the 3,729 laboratory confirmed DR-TB cases that met the inclusion criteria, the majority of the patients had a diagnosis of MDR-TB (80%, n = 2,966). The median age of DR-TB patients was 37 years (IQR 29–45) and approximately half of the patients were male (53% n = 1,992). Majority of the patients had been admitted at a DR-TB hospital for treatment initiation (61% n = 2,293). Just under two thirds (n = 2,435) of DR-TB patients were HIV co-infected and 99% (n = 2,399) of this subpopulation had been initiated onto ART (Table 1).

**Table 1:** Demographic, treatment and health characteristics, by mortality status, of adult patients registered for DR-TB treatment in the Eastern Cape, South Africa, 2011–2013.

Characteristic	Registrations 3,729 (col %)	Deaths 1,445 (row %)	Alive 2,284 (row %)	P-value ^a^
Registration year				
2011	1,314 (35)	540 (41)	774 (59)	0.001
2012	1,102 (30)	438 (40)	664 (60)	
2013	1,313 (35)	467 (36)	846 (64)	
Age in years				
Median (IQR)	37 (29–45)	37 (30–46)	36 (28–45)	<0.001
18–29	993 (27)	337 (34)	656 (66)	
30–44	1,762 (47)	713 (40)	1,049 (60)	
45–59	814 (22)	317 (40)	497 (60)	
60 +	160 (4)	78 (49)	82 (51)	
Sex				
Male	1,992 (53)	766 (38)	1,226 (62)	0.691
Female	1,737 (47)	679 (39)	1,058 (61)	
DR-TB type				
MDR-TB	2,966 (80)	982 (33)	1,984 (67)	<0.001
XDR-TB	763 (20)	463 (61)	300 (39)	
Anti-TB drug history				
New	1,154 (31)	336 (29)	818 (71)	<0.001
History of 1st line drugs	1,998 (54)	782 (39)	1,216 (61)	
History of 2nd line drugs	573 (15)	324 (57)	249 (43)	
Unknown	4 (<1)	3 (75)	1 (25)	
Treatment initiation site				
Community Level	1,341 (36)	329 (25)	1,012 (75)	
DR-TB Hospital	2,293 (61)	1,046 (46)	1,247 (54)	
Unknown	95 (3)	70 (74)	25 (26)	
HIV status				
Negative	1,171 (31)	348 (30)	823 (70)	<0.001
Positive	2,435 (65)	1,038 (43)	1,397 (57)	
Unknown	123 (4)	59 (48)	64 (52)	
On ART	n = 2,435	n = 1,038	n = 1,397	
No	36 (1)	34 (94)	2 (6)	<0.001
Yes	2,399 (99)	1,004 (42)	1,395 (58)	

DR-TB, drug resistant tuberculosis; IQR, inter quartile range; HIV, Human Immunodeficiency Virus; ART, Anti-retroviral therapy;

^a^ P-value calculated using Pearson’s chi square test.

### Description of DR-TB deaths

More than a third of the DR-TB patients died (39%, n = 1,445). Of the 2,966 patients diagnosed with laboratory confirmed MDR-TB, 33% died and 61% of the 763 patients diagnosed with XDR-TB died. Just over half (57%, n = 324) of patients who had previously been treated with second line anti-TB drugs died. A quarter (25%, n = 329) of the patients who were started on DR-TB treatment as outpatients died while just under half (46%, n = 1,046) of those who had started treatment whilst hospitalised died. HIV co-infection amongst DR-TB deaths was 72% (n = 1,038) and of these, 97% (n = 1,004) had been on ART (Table 1).

Among MDR-TB related deaths, males accounted for just over half (56%, n = 548). The majority of MDR-TB patients that died (73%, n = 714) were HIV co-infected and of these 96% (n = 686) had been on ART. Females made up just over half (53%, n = 245) of XDR-TB related deaths and over two thirds (70%, n = 324) of the XDR-TB patients that died were HIV co-infected, and of these 98% (n = 318) had received ART (Table 2).

**Table 2:** Demographic, treatment and health characteristics of drug resistant tuberculosis mortality by drug resistance type, Eastern Cape, South Africa; 2011 to 2013.

Characteristic	MDR-TB deaths n = 982 (column %)	XDR-TB deaths n = 463 (column %)
Age in years		
Median (IQR)	37 (30–46)	36 (29–44)
18–29	212 (21)	125 (27)
30–44	489 (50)	224 (49)
45–59	214 (22)	103 (22)
60 +	67 (7)	11 (2)
Sex		
Female	434 (44)	245 (53)
Male	548 (56)	218 (47)
Previous Drug History		
New	277 (28)	59 (13)
History of 1st line drugs	588 (60)	194 (42)
History of 2nd line drugs	115 (12)	209 (45)
Unknown	2 (<1)	1(<1)
Treatment initiation site		
Community Level	267 (27)	62 (13)
DR-TB Hospital	670 (68)	376 (81)
Unknown	45 (5)	25 (5)
HIV status		
Negative	227 (23)	121 (26)
Positive	714 (73)	324 (70)
Unknown	41 (4)	18 (4)
ART initiation status	n = 714	n = 324
No	28 (4)	6 (2)
Yes	686 (96)	318 (98)

DR-TB, drug resistant tuberculosis; IQR, inter quartile range; HIV, Human Immunodeficiency Virus; ART, Anti-retroviral therapy.

### Risk factors of DR-TB mortality

On multivariable analysis, patients were at higher risk of mortality during the course of DR-TB treatment if they had HIV co-infection (aIRR 3.3, 95%CI 2.9–3.8, for those not on ART and IRR 1.4, 95%CI 1.3–1.5 for those on ART), were older (aIRR 1.7, 95%CI 1.5–2.0, for those ≥ 60 years when compared with <30 year olds), had a diagnosis of XDR-TB (aIRR 1.6, 95%CI 1.5–1.7, when compared to MDR-TB), had prior exposure to anti-TB drugs (aIRR 1.4, 95%CI 1.3–1.5 for those exposed to second line drugs), or had been hospitalised at DR-TB treatment start (aIRR 1.7, 95%CI 1.5–18, compared to those started on ambulatory DR-TB care) (Table 3).

**Table 3:** Factors associated with mortality among adult cases on drug resistant tuberculosis treatment in Eastern Cape, South Africa; 2011 to 2013.

DR TB Characteristic	Univariate Analysis		Multivariable Analysis	
	IRR (95% CI)	P-value ^a^	aIRR (95% CI)	P-value ^a^
HIV ART Status				
Negative	Ref		Ref	
Co-infected on ART	1.4 (1.3–1.5)	<0.001	1.4 (1.3–1.5)	**<0.001**
Co-infected not on ART	3.2 (2.9–3.5)	<0.001	3.3 (2.9–3.8)	**<0.001**
Status unknown	1.6 (1.4–1.9)	<0.001	1.1 (0.8–1.5)	0.546
Age				
18–29	Ref		Ref	
30–44	1.2 (1.1–1.3)	0.001	1.1 (1.0–1.2)	0.107
45–59	1.2 (1.1–1.2)	0.027	1.1 (1.0–1.2)	0.101
60 +	1.4 (1.2–1.7)	<0.001	1.7 (1.5–2.0)	**<0.001**
DR-TB type				
MDR-TB	Ref		Ref	
XDR-TB	1.8 (1.7–1.9)	<0.001	1.6 (1.5–1.7)	**<0.001**
Previous Drug History				
New	Ref		Ref	
History of1^st^ line drugs	1.3 (1.3–1.4)	<0.001	1.2 (1.1–1.3)	0.002
History of 2^nd^ line drugs	1.9 (1.8–2.1)	<0.001	1.4 (1.3–1.5)	**<0.001**
Treatment Initiation Site				
Community	Ref		Ref	
DR-TB Hospital	1.9 (1.7–2.0)	<0.001	1.7 (1.5–1.8)	**<0.001**
Registration Year				
2011	Ref		Ref	
2012	1.0 (0.9–1.0)	0.501	1.0 (1.0–1.1)	0.498
2013	0.9 (0.8–0.9)	0.004	1.1 (1.1–1.2)	0.009

HIV, Human Immunodeficiency Virus; ART, Anti-retroviral therapy; DR-TB, drug resistant tuberculosis; aIRR, adjusted incidence risk ratio,

^a^ P-value calculated using modified Poisson regression.

Further analysis of risk factors was performed by stratifying by type of DR-TB. MDR-TB patients were at higher risk of mortality if they had HIV co-infection (aIRR 3.9, 95%CI 3.3–4.6, for those not on ART and aIRR 1.5, 95%CI 1.4–1.7, for those on ART), were older (aIRR 1.9, 95%CI 1.6–2.3, for those ≥ 60 years), were hospitalised at start of MDR-TB treatment (aIRR 1.7, 95%CI 1.5–1.9), or had prior exposure to second line anti-TB drugs (aIRR 1.4, 95%CI 1.2–1.6). XDR-TB patients had a higher risk of mortality if they had HIV co-infection and were not on ART (aIRR 1.8 95%CI 1.5–2.2) or had been admitted to hospital at start of treatment (aIRR 1.5, 95%CI 1.3–1.8) (Table 4).

**Table 4:** Factors associated with mortality in adults according to drug resistant tuberculosis type in Eastern Cape, South Africa; 2011 to 2013.

DR-TB Characteristic	MDR-TB		XDR-TB	
	aIRR (95% CI)	P-value ^a^	aIRR (95% CI)	P-value ^a^
HIV ART Status				
Negative	Ref			
Co-infected on ART	1.5 (1.4–1.7)	**<0.001**	1.1 (1.0–1.3)	0.062
Co-infected not on ART	3.9 (3.3–4.6)	**<0.001**	1.8 (1.5–2.2)	**<0.001**
Status unknown	1.2 (0.8–1.7)	0.488	1.1 (0.7–1.7)	0.709
Age				
18–29	Ref		Ref	
30–44	1.2 (1.1–1.3)	0.034	1.0 (0.8–1.1)	0.671
45–59	1.2 (1.0–1.3)	0.058	1.1 (0.9–1.2)	0.825
60 +	1.9 (1.6–2.3)	**<0.001**	1.3 (0.9–1.7)	0.179
Treatment Initiation Site				
Community	Ref		Ref	
DR-TB Hospital	1.7 (1.5–1.9)	**<0.001**	1.5 (1.3–1.8)	**<0.001**
Previous Drug History				
New	Ref		Ref	
History of 1^st^ line drugs	1.2 (1.1–1.3)	0.001	1.0 (0.9–1.2)	0.734
History of 2^nd^ line drugs	1.4 (1.2–1.6)	0.001	1.3 (1.1–1.5)	0.018
Registration Year				
2011	Ref		Ref	
2012	1.0 (0.8–1.1)	0.893	1.1 (1.0–1.3)	0.072
2013	1.2 (1.1–1.4)	0.028	1.1 (1.0–1.3)	0.119

MDR-TB, multi drug resistant tuberculosis; XDR-TB, extensively drug resistant TB; HIV, Human Immunodeficiency Virus; ART, Anti-retroviral therapy; aIRR adjusted incidence risk ratios,

^a^ P-value calculated using modified Poisson regression.

## Discussion

Significant risk factors for mortality determined by the study included being HIV co-infected, not on ART, age of 60 years and older, having XDR-TB, prior exposure to second line anti-TB drugs, and being hospitalised at start of DR-TB treatment.

The study determined that HIV co-infection was a risk factor for mortality. HIV co-infected patients not receiving ART were more likely to die during DR-TB treatment. This finding is consistent with a recent nation-wide study conducted in South Africa [10], which determined that HIV co-infected patients either not on ART or with unknown ART status were more likely to die when compared to HIV uninfected patients. HIV co-infected patients who are not on ART become immuno-compromised, resulting in rapid progression of DR-TB leading to death [34, 35]. This risk factor remained significant for MDR-TB related deaths and XDR-TB deaths. The finding among XDR-TB patients’ is consistent with the findings of a study conducted in the same province which found decreased survival times in XDR-TB co-infected patients not on ART [20].

Studies conducted prior to universal access to ART describe HIV co-infection as a risk factor for DR-TB mortality [21, 36]. The increasing availability of ART as well as ART initiation of HIV co-infected DR-TB patients regardless of CD4 count [7, 20], has shown a protective effect on mortality [21, 37]. Recent studies have reported no difference in survival between HIV co-infected patients on ART and HIV uninfected patients [20, 38]. Our study was conducted in the post ART availability era [7, 20] and thus we did not expect being HIV co-infected on ART to be a significant risk factor for mortality, nor for it to account for a large proportion of deaths. The study did not assess the effect of CD4 count [18, 39] or timing of ART initiation on mortality [40, 41] due to data limitations. ART status was captured as a static and not a time varying event and thus did not reflect timing of ART initiation as well as defaulting ART [39]. Though our study did not explore this, studies on timing of ART initiation [18, 41] have shown that delaying ART increases the risk of mortality, and this offers a probable explanation for mortality among co-infected patients who received ART co-therapy.

CD4 count is a direct indicator of HIV related immune depletion, as well as near-term risk of opportunistic morbidity and mortality. Together with viral load, CD4 count is used to stage HIV infection. An immunologic reserve (CD4 count) of less than 200 cells per cubic millimetre (mm³) is a threshold below which fatal opportunistic infections become common [42]. An Eastern Cape study conducted among 108 co-infected XDR-TB patients, started on treatment between 2006 and 2008, described the median CD4 count at treatment start as 215 cells/mm³ [20], with some counts as low as 40 cells/mm³. Another South African study described a median CD4-cell counts of 273 cells/mm³ at DR-TB treatment start for patients initiating therapy between 2002 and 2006 [43]. The low to borderline CD4 counts at ART initiation suggests immune-deficiency among co-infected patients, resulting in poor prognosis at ART initiation. Low CD4 count may explain the increased risk of mortality observed among co-infected patients receiving co-therapy in this study. Though previous studies have given probable explanations for mortality in HIV co-infected patients, there is need to explore this risk factor in the Eastern Cape Province, as it accounts for over two thirds of patients that demised in this setting, with high ART initiation among co-infected patients.

Our study determined that being aged 60 years or older was a significant risk factor for mortality. This finding was consistent with Schnippel and colleagues [10] findings on risk factors of rifampicin resistant TB mortality. They determined that age of 60 years and above was an independent risk factor for mortality [10]. Increasing age is associated with increasing co-morbidities [44] as well as general physical deterioration. Comorbidities such as malignancy and diabetes, which increase in prevalence as age increases, have been shown to be risk factors contributing to DR-TB deaths [25]. Our study did not assess contribution of comorbidities other than HIV co-infection on DR-TB mortality.

This study also determined that previous exposure to anti-TB drugs increased the probability of death when compared to new patients; this remained significant in the stratified analysis of MDR- and XDR-TB deaths. This finding is consistent with a South African study [10], which determined that history of anti-TB treatment was a risk factor for mortality. Dheda and colleagues [43] observed similar findings among XDR-TB patients. Another study conducted in KwaZulu-Natal province, South Africa [18] found that risk of death among DR-TB patients was highest with increased degree of drug resistance. One study set in Peru determined that risk of death increased with increased number of previous TB episodes [25]. Patients with prior exposure to anti-TB treatment have an increased chance of developing additional resistance [25, 43] to anti-TB drugs, which limits the number of effective drugs available for inclusion in a treatment regimen. Such ineffective regimens will result in poor survival [39].

Our study determined that patients who were hospitalised at start of treatment were more likely to have died when compared to patients who were initiated onto TB treatment as outpatients. Being hospitalised at start of treatment was a risk factor for mortality as patients who had poorer prognoses may have been prioritised for hospital admission whilst patients with more favourable prognosis were likely to have started treatment in the community. This is in line with the national guidelines that prioritises hospital admission for clinically unstable MDR-TB patients, patients with extensive disease and XDR-TB patients [16, 32]. This study could not determine the proportion of patients initiated in the community that were subsequently admitted into DR-TB hospitals, thus treatment initiation in the community was not strictly synonymous with decentralisation of MDR-TB services. Better treatment outcomes associated with decentralisation of services have been attributed to positive psycho-social factors such as familiar environment, minimal disruption of daily lives, as well as availability of family support [31, 32]. Along with possible early DR-TB treatment start, these psycho-social factors may assist in explaining why being initiated in community was protective for mortality. It is important to note that this risk factor may also be a marker for disease severity and prognosis as well as co-morbidities that was unmeasured due to data limitations.

Our study had some limitations that should be considered when interpreting the findings. Firstly, we utilised data that was collected for routine surveillance purposes. Incompleteness of data limited our ability to explore timing of events in relation to mortality. Routinely reported information is not always structured to answer research questions and as such, an in depth mortality study may highlight aspects not explored by this study. Secondly, exclusion of mono, poly and not confirmed resistant tuberculosis from the study may have introduced bias.

Possible misclassification of deaths from other non DR-TB causes could have resulted in over-estimation of DR-TB deaths. Misclassification could have led to increased statistical power during multivariable analysis that exaggerated the relationship between death and risk factors. According to the national policy, DR-TB death is defined as a death that occurs during DR-TB treatment irrespective of cause of death [7]. Circumstances surrounding death were infrequently captured and, as such, reclassification of deaths could not be done. The study may also under-estimate mortality as MDR- or XDR-TB cases may die before diagnosis or treatment start.

Effects of programmatic developments such as roll-out of GXP testing [45] may have been masked as the study did not explore time series analysis of events. Nationwide rollout of the test began in 2011 [46] and GXP testing may have resulted in a decrease in the time from diagnosis to treatment initiation. GXP is a WHO approved rapid diagnostic test [1] with a turn-around time of 2 hours [46]. However, such effects were managed by incorporating year of registration in the analysis, and the large sample size in this study maintained the power of the study.

## Conclusion

Though no new findings were determined, this province wide study provides baseline understanding of the epidemiology as well as risk factors for both MDR-TB and XDR-TB mortality in the Eastern Cape. Being HIV co-infected and not on ART, age of 60 years and above, and being admitted for DR-TB treatment initiation were identified as significant risk factors for DR-TB mortality in adults. Continued strengthening of the integration of TB/ HIV services with emphasis on HIV testing and counselling of DR-TB patients and provision of ART for all co-infected patients may assist in reducing DR-TB mortalities in the Eastern Cape. We recommend further studies using more robust data collection and data analysis methods in order to further explore the incidence and associated risk factors of DR-TB mortality, in the province.
